# Ascorbic Acid Preconditioning Effect on Broccoli Seedling Growth and Photosynthesis under Drought Stress

**DOI:** 10.3390/plants11101324

**Published:** 2022-05-17

**Authors:** Mason T. MacDonald, Rajeswari Kannan, Renuga Jayaseelan

**Affiliations:** Faculty of Agriculture, Dalhousie University, Bible Hill, NS B2N 5E3, Canada; rj938452@dal.ca (R.K.); rn598954@dal.ca (R.J.)

**Keywords:** antioxidant, *Brassica oleracea*, chlorophyll index, membrane injury, stomatal conductance, water deficit, water use efficiency

## Abstract

Drought is an abiotic stress that decreases crop photosynthesis, growth, and yield. Ascorbic acid has been used as a seed preconditioning agent to help mitigate drought in some species, but not yet in broccoli (*Brassica oleracea* var. *italica*). The objective was to investigate the effect of ascorbic acid on growth, photosynthesis, and related parameters in watered and drought-stressed broccoli seedlings. A 2 × 4 factorial experiment was designed where stress (watered or drought) was the first factor and ascorbic acid preconditioning (untreated, 0 ppm, 1 ppm, or 10 ppm) was the second factor. Positioning within the greenhouse was included as a blocking factor and the experiment was replicated three times. All seedlings were watered for 8 weeks and then half had water withheld for 7 days to impose drought while the other half continued to be watered. Ascorbic acid preconditioning increased shoot dry mass, root dry mass, water use efficiency, and photosynthesis in all seedlings while also increasing chlorophyll, relative water content, and leaf area in droughted seedlings. Ascorbic acid preconditioning also decreased membrane injury in droughted seedlings to the point that membrane injury was not significantly different than the watered control. There was strong evidence to support ascorbic acid as a successful seed preconditioning agent in watered and droughted broccoli.

## 1. Introduction

Broccoli is a member of the Brassicaceae family that grows best at 13–20 °C [1]. Broccoli grows 60–90 cm tall, forms a branching green stalk, and has dense flower buds that represent the commonly consumed organ [2]. The market for broccoli has expanded significantly in the latter half of the 20th century, with a yield that has increased to approximately 26 million tons globally [3]. The increased popularity of broccoli is largely due to its many desirable nutrients, antioxidants, and other bioactive compounds [2,4]. Broccoli is considered a cool season crop and, like many crops, is vulnerable to several abiotic stresses that might limit productivity [5].

Drought is one abiotic stress critical to photosynthesis and crop yields [6]. Drought may occur because of insufficient rainfall, increased evapotranspiration, or increased salinity in soils [7,8]. Stomata close rapidly after the onset of drought to conserve water, but this is accompanied by reduced CO_2_ absorption, photosynthesis (Pn), and growth [9,10]. A reduction in CO_2_ absorption causes an imbalance between electron excitation and utilization during photosynthesis, which in turn increases the production of reactive oxygen species (ROS) [11]. ROS serve, in part, as a physiological signal indicative of abiotic stress, but also cause significant damage to cellular membranes and biochemicals [12]. 

Drought stress can affect a variety of agricultural species. Drought decreased Pn and increased ROS production in several wheat cultivars [13], decreased Pn in apple trees [14], decreased Pn in several forestry species [15], and decreased Pn and yields in several Brassica species [16,17]. Even mild drought can adversely affect broccoli yields where a 20% and 40% decrease in irrigation resulted in 29% and 41% lower broccoli yields, respectively [18]. Closely related crops, such as cauliflower, also had reduced germination, shoot length, root length, curd growth, and dry mass (DM) due to drought [5]. The cumulative effects of drought have reduced global crop yields by 10% from 1964 to 2007 [19]. Drought-induced crop losses of only maize, rice, soy, and wheat from 1983 to 2009 were estimated at USD 166 billion [20]. 

Seed preconditioning or priming is one of the many technologies available to help mitigate drought stress. Seed preconditioning involves incubating seeds with a seed preconditioning agent (SPA) to induce benefits in developing seedlings and mature plants [21]. Many compounds have been used successfully as SPAs. Salicylic acid, acetylsalicylic acid, and glycinebetaine promoted germination in carrots [22], 5-hydroxybenzimidazole increased yields and stress resistance in tomatoes [23], and pyroligneous acid increased yields in rice [24]. Several naturally occurring antioxidants, such as lycopene, β-carotene, and ascorbic acid (AsA), also increased tomato dry mass and photosynthesis when used as SPAs [25].

Seed preconditioning with AsA is of particular interest because it is inexpensive, readily accessible, and has benefitted several crops. Seed preconditioning with 1 ppm and 10 ppm AsA increased tomato shoot dry mass by 40%, leaf area by 50%, and increased Pn by 223% compared to a control [25]. Preconditioning with 50 ppm AsA increased wheat grain yields by approximately 26% [26]. Finally, preconditioning with 10 ppm and 20 ppm AsA increased rice germination and dry mass [27]. However, there is no literature on the effect of AsA preconditioning on broccoli. 

We hypothesize that AsA will promote drought stress tolerance in broccoli. In other species, varieties with increased drought tolerance typically presented with delayed decreases in Pn, higher biomass and leaf area, and decreased membrane injury [13,14,15,25]. Thus, it is predicted that AsA-preconditioned broccoli would have similar responses. The specific objectives of this research are to investigate the effect of AsA on growth, photosynthesis, and related parameters in watered and drought-stressed broccoli seedlings.

## 2. Results

### 2.1. Soil Moisture

**Figure 1:**
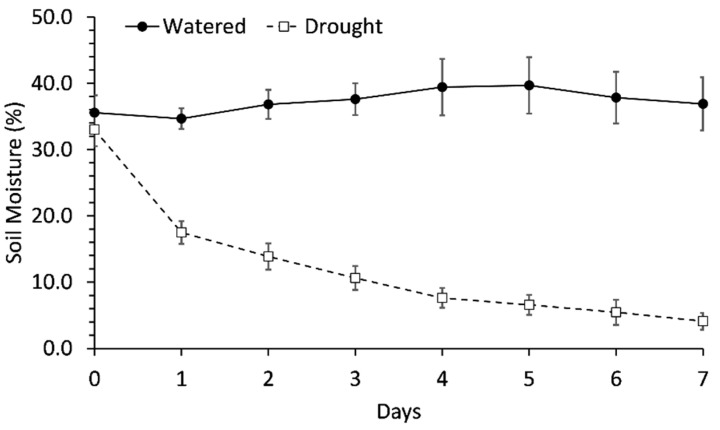
Soil moisture over time in pots during the last week of the experiment. Watered pots continued to receive water each day while water was completely withheld from droughted pots. Each data point represented the mean of 36 pots in each treatment. Error bars indicate standard error.

Soil moisture was maintained at 38.5 ± 1.5% in all treatments throughout the first 8 weeks of the experiment and was not significantly different between drought and watered treatments until the imposition of drought. However, average soil moisture decreased below 20% after withholding water for 1 day and then gradually decreased to 4.1% after water was withheld for 1 week (Figure 1). Though there was a significant difference in soil moisture between watered and droughted treatments at the end of the experiment (Figure 1), there were no differences in soil moisture between any preconditioning treatments.

### 2.2. Biomass

**Figure 2:**
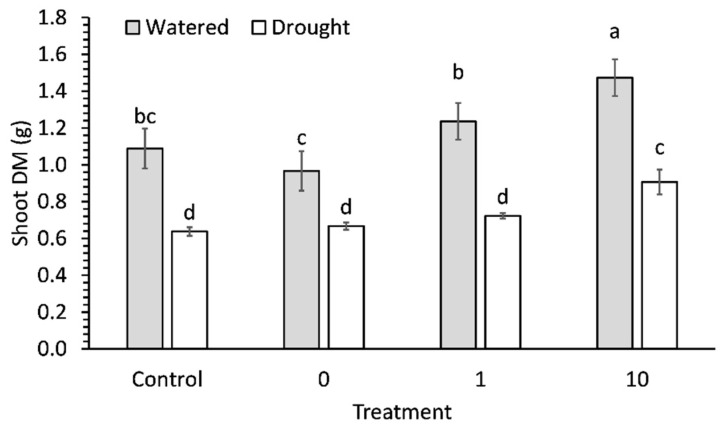
Shoot biomass in 9-week-old broccoli seedlings that were well watered or droughted for 1 week. Each bar represents a mean and error bars represent standard error calculated from 9 replicates. Means with different letters are significantly different (*p* < 0.05) as determined by Tukey’s honestly significant difference.

**Figure 3:**
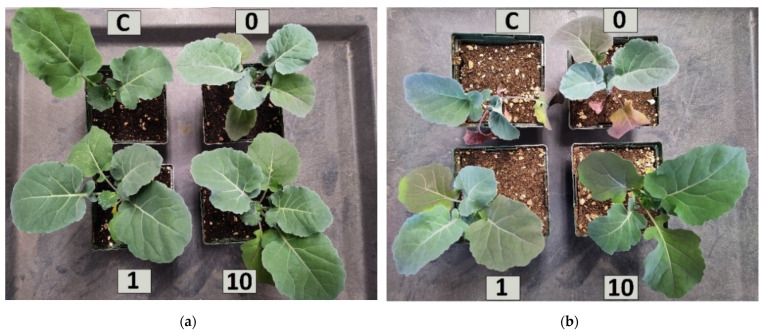
Overhead view of 9-week-old broccoli seedlings that were (**a**) watered and (**b**) deprived of water for 1 week. Control seedlings (C) were provided no seed preconditioning while others were provided 0 ppm, 1 ppm, or 10 ppm of ascorbic acid preconditioning.

Drought stress significantly (*p* < 0.001) decreased shoot DM by approximately 39% compared to watered plants and the second was that preconditioning seeds with 10 ppm significantly (*p* < 0.001) increased shoot DM when compared to a control (Figure 2). The 10 ppm-preconditioned watered seedlings had 35% and 52% higher shoot DM than non-preconditioned or water-preconditioned seedlings, respectively. Further, the 10 ppm-preconditioned droughted seedlings had 42% and 36% higher shoot DM than non-preconditioned or water-preconditioned seedlings, respectively. Differences in shoot size were visually apparent (Figure 3).

**Figure 4:**
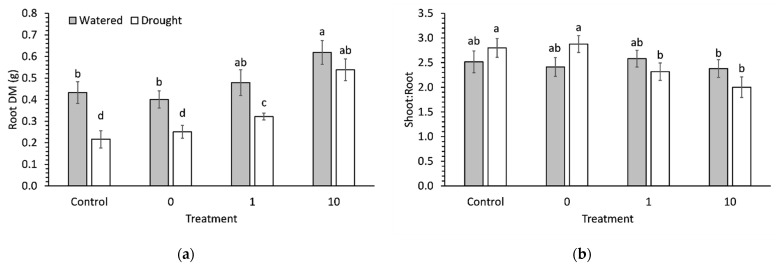
(**a**) Root biomass and (**b**) shoot:root ratio in 9-week-old broccoli seedlings which were watered or deprived of water for 1 week. Each bar represents a mean and error bars represent standard error calculated from 9 replicates. Means with different letters are significantly different (*p* < 0.05) as determined by Tukey’s honestly significant difference.

AsA preconditioning had a significant (*p* = 0.007) effect on root DM. Both 1 ppm and 10 ppm preconditioning significantly (*p* = 0.007) increased root DM by 49% and 149% compared to the control, respectively. Only the 10 ppm AsA preconditioning increased root DM in watered seedlings compared to controls (Figure 4a). The increase in DM due to preconditioning was more pronounced in roots than shoots in AsA-preconditioned seedlings, which resulted in a significant decrease in shoot:root ratio of droughted seedlings compared to the control (Figure 4b). There were no differences in the shoot:root ratios of watered seedlings due to preconditioning.

### 2.3. Photosynthetic Measurements

**Figure 5:**
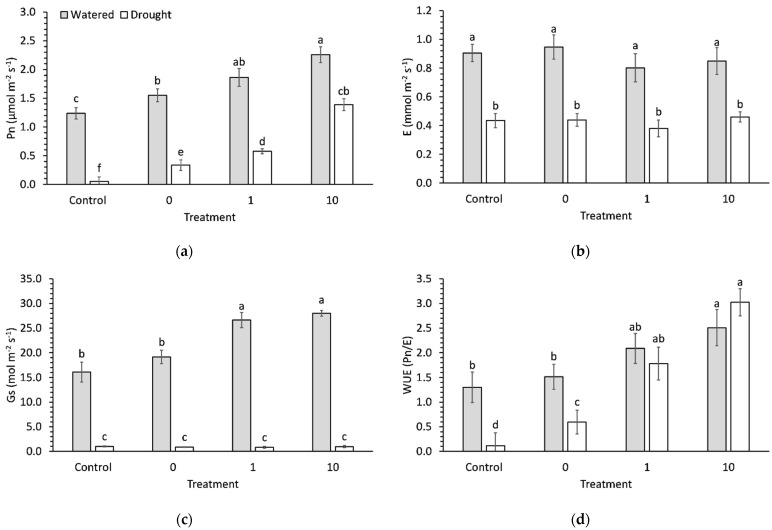
(**a**) Photosynthesis, (**b**) transpiration, (**c**) stomatal conductance, and (**d**) water use efficiency in 9-week-old broccoli seedlings that were well watered or droughted for 1 week. Each bar represents a mean and error bars represent standard error calculated from 9 replicates. Means with different letters are significantly different (*p* < 0.05) as determined by Tukey’s honestly significant difference.

Pn was significantly (*p* < 0.001) decreased by 68% due to drought compared to watered seedlings (Figure 5a). Ascorbic acid preconditioning significantly (*p* < 0.001) increased Pn in both watered and droughted seedlings. Preconditioning with 10 ppm ascorbic acid increased Pn by 83% compared to a control in watered seedlings. The droughted control had virtually ceased Pn, but droughted seedlings preconditioned with 10 ppm ascorbic acid maintained photosynthetic rates that were not significantly different than watered controls (Figure 5a).

Transpiration (E) and stomatal conductance (Gs) were significantly (*p* < 0.001) decreased by 51% and 96%, respectively, due to drought compared to watered seedlings (Figure 5b,c). AsA preconditioning had no significant impact on E (Figure 5b), though Gs was significantly higher in both AsA treatments in watered seedlings (Figure 5c). Consequently, there was a significant (*p* = 0.03) interaction effect between stress and preconditioning on WUE. Preconditioning with 10 ppm ascorbic acid increased WUE by almost 2-fold in watered seedlings, but by 26-fold in droughted seedlings compared to their respective controls (Figure 5d).

### 2.4. Leaf Measurements

**Figure 6:**
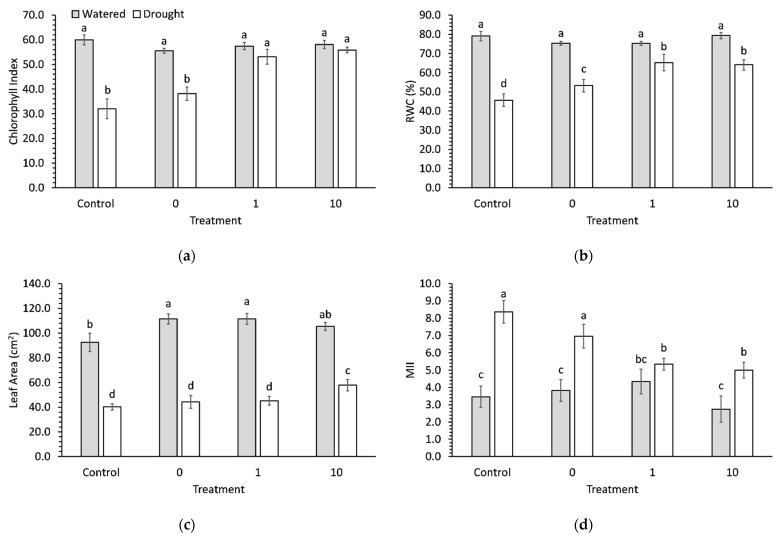
(**a**) Chlorophyll index, (**b**) relative water content, (**c**) leaf area, and (**d**) membrane injury index in 9-week-old broccoli seedlings that were well watered or droughted for 1 week. Each bar represents a mean and error bars represent standard error calculated from 9 replicates. Means with different letters are significantly different (*p* < 0.05) as determined by Tukey’s honestly significant difference.

There was a significant (*p* < 0.001) interaction between stress and preconditioning on chlorophyll index (Figure 6a). Drought decreased chlorophyll index by 47% and 31% in seedlings that were not preconditioned or preconditioned with water only, respectively, compared to watered seedlings. However, drought did not affect chlorophyll index in seedlings preconditioned with 1 ppm or 10 ppm ascorbic acid.

There was a significant (*p* = 0.028) interaction between stress and preconditioning on relative water content (RWC) (Figure 6b). Drought decreased RWC by 42% and 29% in seedlings that were not preconditioned or preconditioned with water only, respectively, compared to watered seedlings. Drought also decreased RWC in preconditioned seedlings, but only by 13% and 19% in seedlings preconditioned with 1 ppm and 10 ppm ascorbic acid, respectively, compared to watered seedlings.

Both stress and preconditioning had a significant (*p* < 0.001 and *p* = 0.012) effect on leaf area (Figure 6c). Leaf area was significantly higher than the control due to water or 1 ppm ascorbic acid preconditioning in watered seedlings but was significantly higher than the control due to only 10 ppm ascorbic acid preconditioning in droughted seedlings. Drought generally decreased leaf area by an average of 56% in all seedlings.

There was a significant (*p* < 0.001) interaction between stress and preconditioning on membrane injury index (MII) (Figure 6d). There was no difference in MII between preconditioning treatments in watered seedlings. However, MII was 36% and 40% lower in 1 ppm- and 10 ppm-preconditioned droughted seedlings, respectively, compared to a control. MII in droughted seedlings that had been preconditioned with 10 ppm ascorbic acid was not significantly lower than any watered seedlings.

## 3. Discussion

AsA increased Pn, WUE, and shoot DM in watered broccoli seedlings, which was comparable to AsA effects on tomatoes [25]. Pn appears to be a key response since Pn would influence both shoot DM and WUE; increased Pn would result in increased CO_2_ fixation and an increase in Pn without any change in E would directly correspond to WUE. Although this experiment did not grow plants to maturity, it seems plausible that AsA preconditioning could promote broccoli yields in a similar manner to previous SPAs [23].

AsA preconditioning affected more response variables in drought-stressed seedlings than watered seedlings. Pn, WUE, shoot DM, root DM, leaf area, chlorophyll, and RWC content all increased while MII decreased in AsA preconditioned seedlings exposed to drought. As above, this is comparable to previous results with tomato seedlings [25]. Additionally, as above, the increase in Pn likely explains increases in shoot DM, root DM, and WUE. 

Since PM, DM, and WUE were higher in all AsA-preconditioned seedlings compared to their respective controls, these responses in droughted seedlings are not specific indicators of drought stress tolerance. However, chlorophyll, RWC, leaf area, and MII were negatively impacted by drought but largely mitigated by AsA preconditioning, which is indicative of increased stress tolerance. Maintenance of chloroplasts due to AsA preconditioning would result in improved light capture and energy conservation, which would help explain why Pn remained so high during drought [28]. Further, AsA-preconditioned seedlings also had higher RWC during drought, which would improve biosynthesis and chlorophyll proteins and, consequently, Pn [14,29].

The specific mechanism through which AsA confers benefits to developing seedlings remains unclear. One explanation is that since watered seedlings were larger, perhaps seedlings were more apt to survive drought. Several studies report either larger unstressed plants or accelerated germination/emergence due to AsA [22,23,26,30]. A second explanation is that the increased root DM or decreased shoot:root ratio allowed for AsA-preconditioned plants to access more water during drought [25]. Root access of water seems unlikely though because all seedlings were grown in pots and soil moisture never differed among the preconditioned treatments. A third explanation is that, as an antioxidant, AsA is protecting cellular membranes from stress-induced oxidative damage. Certainly, a significant decrease in MII supports the concept of membrane protection, which would include chloroplast membranes and help explain why Pn was maintained during drought. However, a direct antioxidant effect seem unlikely since seeds were treated several weeks before imposed stress, Pn was increased in watered seedlings, and there are instances where other potent antioxidants have had no effect on MII when used as SPAs [25].

For an SPA to provide any benefit to developing or mature plants, it seems likely there must be some change directly within seeds that persists as plants develop. Several SPAs induced new proteins that were not present in untreated or water preconditioned seeds [25]. Further, some SPAs seem to induce an epigenetic effect since next generation seedlings grown from preconditioned parents had similar benefits as their parents [23]. Two previous studies support the concept of AsA-induced proteins; there was an increase in soluble proteins and protease in AsA-preconditioned rice [27] and increased protease, peroxidase, and superoxide dismutase in AsA-preconditioned wheat [26]. Further work is needed to identify which induced proteins are increasing photosynthesis, growth, and/or drought tolerance.

Changes in osmoregulation of droughted seedlings must be considered as a possible mechanism for AsA preconditioning-induced drought tolerance. It is well established that osmoregulation through the accumulation of sugars, ions, amino acids, or other metabolites can promote drought tolerance [13,28,31]. Exogenously applied AsA increased the uptake of several cations and osmolytes proline and glycinebetaine, which increased stress tolerance [32]. Seed priming with other SPAs increased the concentration of several osmolytes that helped mitigate stress [33,34] and it stands to reason that AsA would have a similar effect as an SPA.

## 4. Materials and Methods

### 4.1. Experimental Design

This experiment was designed with two factors of interest and one blocking factor. The first factor of interest was seed preconditioning with 4 levels, where seeds were either not preconditioned (control), preconditioned with water only (0 ppm), preconditioned with 1 ppm AsA, or preconditioned with 10 ppm AsA. The second factor was stress level which had 2 levels. All broccoli seedlings were grown and watered for 8 weeks and then half continued to be watered while the other half were deprived of water for 1 week. The blocking factor was based on location within a greenhouse with seedlings placed in 3 different positions. The experiment was replicated 3 times, which required 72 broccoli seedlings overall with 1 seedling per pot.

### 4.2. Seed Preconditioning

A 1000 ppm stock solution of AsA (Sigma-Aldrich, Oakville, ON, Canada) was made in deionized water. The stock solution was diluted to 10 ppm and 1 ppm while deionized water only was used as the 0 ppm AsA treatment. Each of the three preconditioning treatments was transferred to a 250 mL flask. Exactly 50 seeds were added to each flask and placed in a G24 Environmental incubator Shaker (NB Scientific Co., Inc., Woodbridge Township, NJ, USA) at 150 rpm and 20 °C. After 24 h, flask contents were poured through a sieve screen and seeds were dabbed dry. Control seeds were not preconditioned at all.

### 4.3. Growing Conditions

Seeds were grown in a greenhouse starting 11 January 2022 in 10 cm pots using Promix (Halifax Seed, Halifax, NS, Canada) as substrate then moved to a greenhouse. There were three trays in different locations within the greenhouse that served as experimental blocks. Each tray was randomly assigned six pots of each preconditioning treatment, for a total of 24 pots per tray. Pots were randomly arranged on each tray.

All seedlings were grown for 8 weeks under the same conditions. The greenhouse was set at 20 °C/15 °C day/night temperatures. All seedlings were watered daily and provided 200 ppm of 20-20-20 fertilizer, 5 ppm of iron chelate, and 2 ppm of micro-chelate (all from Halifax Seed, Halifax, NS, Canada) once per week for the first 8 weeks. However, after the 8th week, half the pots were deprived of water for 1 week to simulate drought stress. Seedlings in the watered treatment were not provided fertilizer for this final week. 

All response variables were measured at the end of the 9th week except soil moisture. Soil moisture was measured daily throughout the experiment using an HH2 Moisture Meter (Delta-T Devices, Cambridge, UK) to ensure all pots were being maintained at similar soil moisture and to evaluate the difference in soil moisture during drought. 

### 4.4. Photosynthetic Measurements

Pn, E, and Gs were measured using an LCA-4 Gas Exchange System (ADC Bioscientific, Hoddesdon, UK). The procedure was modified from [25] so that each measurement was taken three times, once after 30 s, 60 s, and 90 s. Pn, E, and Gs were each reported as the average of those three measurements. WUE was calculated as a ratio of Pn:E. Gas exchange measurements were taken on a cloudy day where light intensity in the greenhouse was 238 ± 5.7 μmol m^−2^ s^−1^ provided via ambient light as determined by 3 measurements per block. The temperature was 20 °C with a relative humidity 60% (vapor pressure deficit = 0.94 kPa).

### 4.5. Intact Leaf Measurements

Chlorophyll index was measured with a Minolta SPAD 504 meter (Konica Minolta, Ramsey, NJ, USA). The newest fully expanded leaf was placed in the sensor and the instrument measured the transmittance at wavelengths of 650 nm and 940 nm [35]. Results were averaged from five readings from each replicate.

Leaf area was measured using ImageJ processing and analysis software (National Institutes of Health, Bethesda, MD, USA). A scaled white paper backboard was placed around seedling stems at soil level and overhead photos were taken of each seedling. Each photo had a scale set before conversion into a binary image. The entire overhead leaf canopy was selected using the flood fill tool to determine area.

### 4.6. Detached Leaf Measurements

Relative water content (RWC) was measured in all seedlings at the end of the 9th week. One leaf (approximately 0.6 g) was cut from each seedling, weighed for fresh mass (M_f_), and then placed in deionized water to reach full turgidity. After 24 h, the leaves were removed from water, surface moisture was removed by dabbing with tissue paper, and leaves were weighed for turgid mass (M_t_). Leaves were then dried at 90 °C for 24 h and then weighed again for dry mass (M_d_). The following calculation from [36] was used:

(1) $$\mathrm{RWC}=\frac{M_{f}-M_{d}}{M_{t}-M_{d}}\times 100$$

MII uses the percentage of electrolyte leaked into solution to quantify membrane integrity [37]. Centrifuge tubes were filled with 30 mL of deionized water and were allowed to adjust to room temperature (25 °C). The electrical conductivity of the deionized water (EC_w_) alone was measured using a CDM 2e Conductivity Meter (Bach-Simpson, London, ON, Canada). Afterward, 1 leaf (approximately 0.4 g) was removed from each seedling and completely submerged in a centrifuge tube. The tubes were sealed and left at room temperature for 24 h. Initial conductivity (EC_0_) was measured to quantify the electrolytes leached into solution. Sealed tubes were then placed in a forced-air oven for 4 h at 90 °C to kill tissues and then cooled to room temperature. Final conductivity measurements (EC_f_) were taken after equilibrating to 25 °C to determine maximum leakage. MII was then calculated using the following formula:

(2) $$\frac{{\mathrm{EC}}_{0}-{\mathrm{EC}}_{w}}{{\mathrm{EC}}_{f}-{\mathrm{EC}}_{w}}\times 100$$

### 4.7. Biomass Measurements

Entire seedlings were removed from their pots and then separated into shoot and root. Roots were cleaned of soil then roots and shoots were each dried in a hot air over at 80°C for 48 h. Shoots and roots were weighed to determine DM. The leaf DMs from Section 4.6 were added to their respective shoot DM, so that final values were indicative of the entire shoot.

### 4.8. Statistical Analysis

Data were submitted to Minitab 19 (Minitab LLC., Centre County, PA, USA) to analyze as a general linear model. The model included main and interactive effects of stress level and preconditioning treatments as well as the experimental block. Statistical assumptions of homogeneity, normality, and independence were verified. Multiple means comparison was completed using Tukey’s honestly significant difference at 5% significance.

## 5. Conclusions

These results support our original hypothesis that AsA preconditioning promotes growth and drought stress tolerance in broccoli seedlings. Pn, root DM, shoot DM, and WUE efficiency were increased in watered and drought-stressed seedlings. Root DM, chlorophyll, MII, RWC, and leaf area were all also improved in drought-stressed seedlings. Improved membrane protection association with chlorophyll index and MII combined with maintained RWC are indicative of drought stress tolerance, which contributed to positive effects of Pn and growth.

From a practical perspective, this research represents a viable SPA technology that could be important for industry. AsA is a relatively inexpensive SPA (CAD~0.20/g) and the required concentration of 10 ppm is relatively low. The cost of preconditioning 50 seeds with AsA based on our methodology was only US $0.0005. Yet, AsA preconditioning improved shoot DM and Pn by up to 52% and 83%, respectively, which would have tremendous value for commercial operations. Further research would be required to determine the effect on yield.

From a physiological perspective, this research presents further evidence of additional roles of AsA in plant physiology. Exogenous application of AsA to seeds has induced several positive effects in developing plants. It seems likely that specific proteins are triggered within the developing broccoli seedlings that protect chloroplasts, membranes, and photosynthesis. AsA represents the impetus for these physiological changes, but there are several other steps that remain unknown to understand the entire physiological mechanism.

## Data Availability

Not applicable.
